# Supplementary figures and images for: High-resolution 3D imaging and topological mapping of the lymph node conduit system
Source: PLoS Biol. 2019 Dec 19;17(12):e3000486. doi: 10.1371/journal.pbio.3000486 (PMC6922347; doi:10.1371/journal.pbio.3000486)

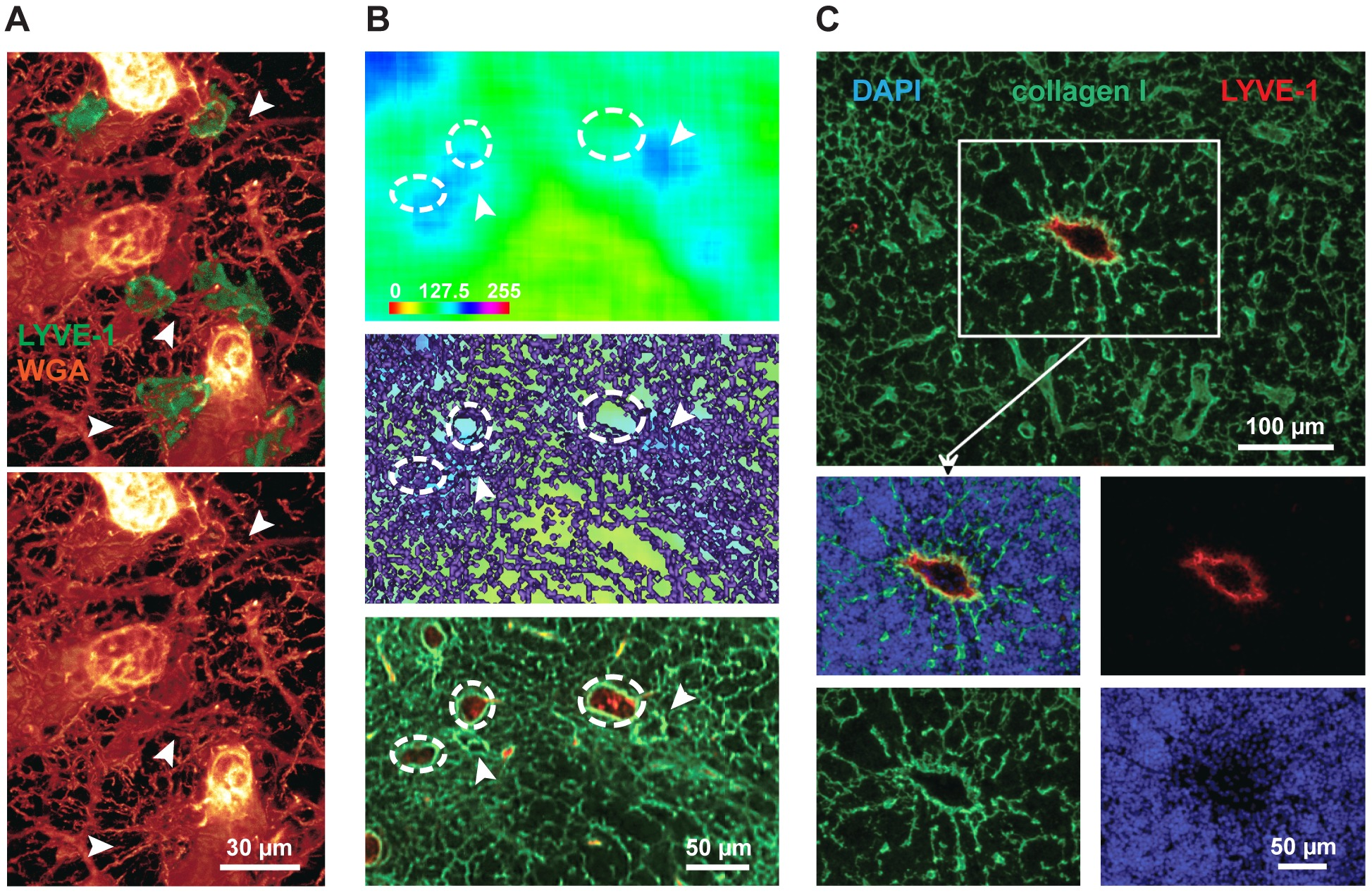

Supplement: S1 Fig — Related to Fig 1. High-resolution confocal images of LN conduits, blood vessels (both co-labeled via perfusion in red), and lymphatic sinuses (LYVE-1, green) display conduits fusing into sinuses in close proximity to large blood vessels (arrowheads, panel A). In a moving average display (upper panel) generated from 3D LN images with WGA-labeled lymphatic channels that color codes pixel density in a rainbow spectrum, regions with a high density of conduits appear blue (B). These blue regions do not overlap with the location of blood vessels, seen as gaps in the conduit mesh (dashed circles), as can be taken from an overlay of the moving average image with the corresponding section of the conduit network image (B, middle). Instead, regions with high density of conduit stain show an association with lymphatic sinuses (arrowheads) that stain brightly with WGA (green) and lack a vascular core (red, B, lower). In multicolor fluorescent images of an immuno-labeled LN section, several collagen I+ conduits concentrate on a LYVE-1+ lymphatic sinus (C). LN, lymph node; LYVE-1, lymphatic vessel endothelial receptor 1; WGA, wheat germ agglutinin. (TIF) [file pbio.3000486.s001.tif]

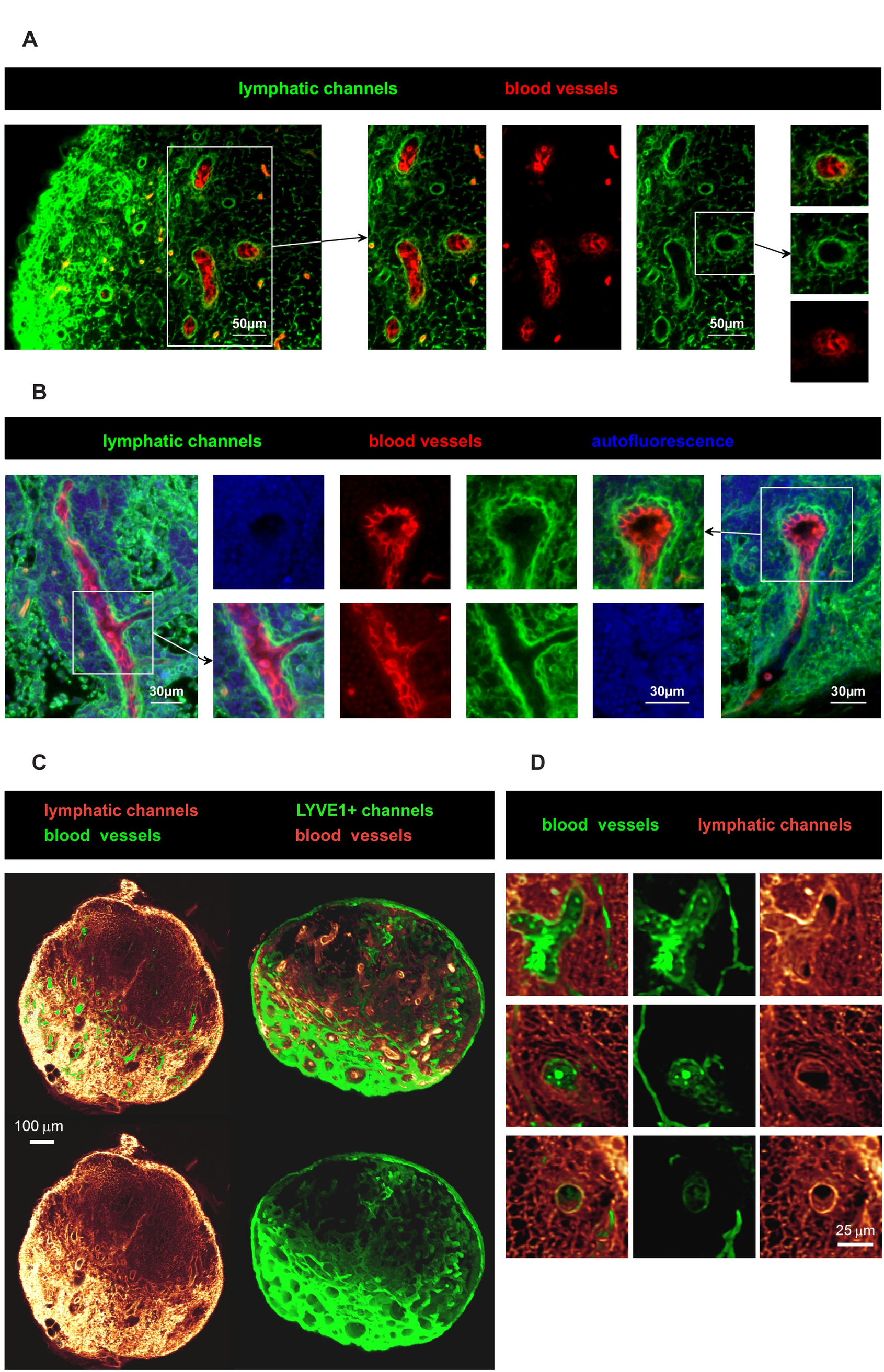

Supplement: S2 Fig — Related to Fig 2. Murine LNs were perfused with fluorescently tagged WGA and 2,000 kDa dextran to label the blood vasculature (red) and locally injected with fluorescently tagged WGA to label the lymphatic channels including conduit passageways (green). Multicolor fluorescent images of 2 μm LN sections show LN blood vessels are surrounded by sleeves continuous with the conduits (A). Close-up fluorescent images confirm the close juxtaposition of blood vessel endothelium (red) enclosed by a cell layer stained with lymph-borne WGA (green) against the background of autofluorescent cell bodies (blue, B). 3D reconstructed images of an LN volume image generated by EVIS imaging (at 1 μm pixel resolution, C) visualize the overall arrangement of the WGA-labeled channels including conduits and lymphatic vessels (red glow, left panel), the latter of which also stain positively for LYVE-1 (green, right panel), against the dense network of blood vessels weaving through the LN. Close-up images of 20 μm optical sections of a LN volume image illustrate how the conduit sleeves (red glow) fully enclose blood vessels (green, D). EVIS, extended-volume imaging system; LN, lymph node; LYVE-1, lymphatic vessel endothelial receptor 1; WGA, wheat germ agglutinin. (TIF) [file pbio.3000486.s002.tif]

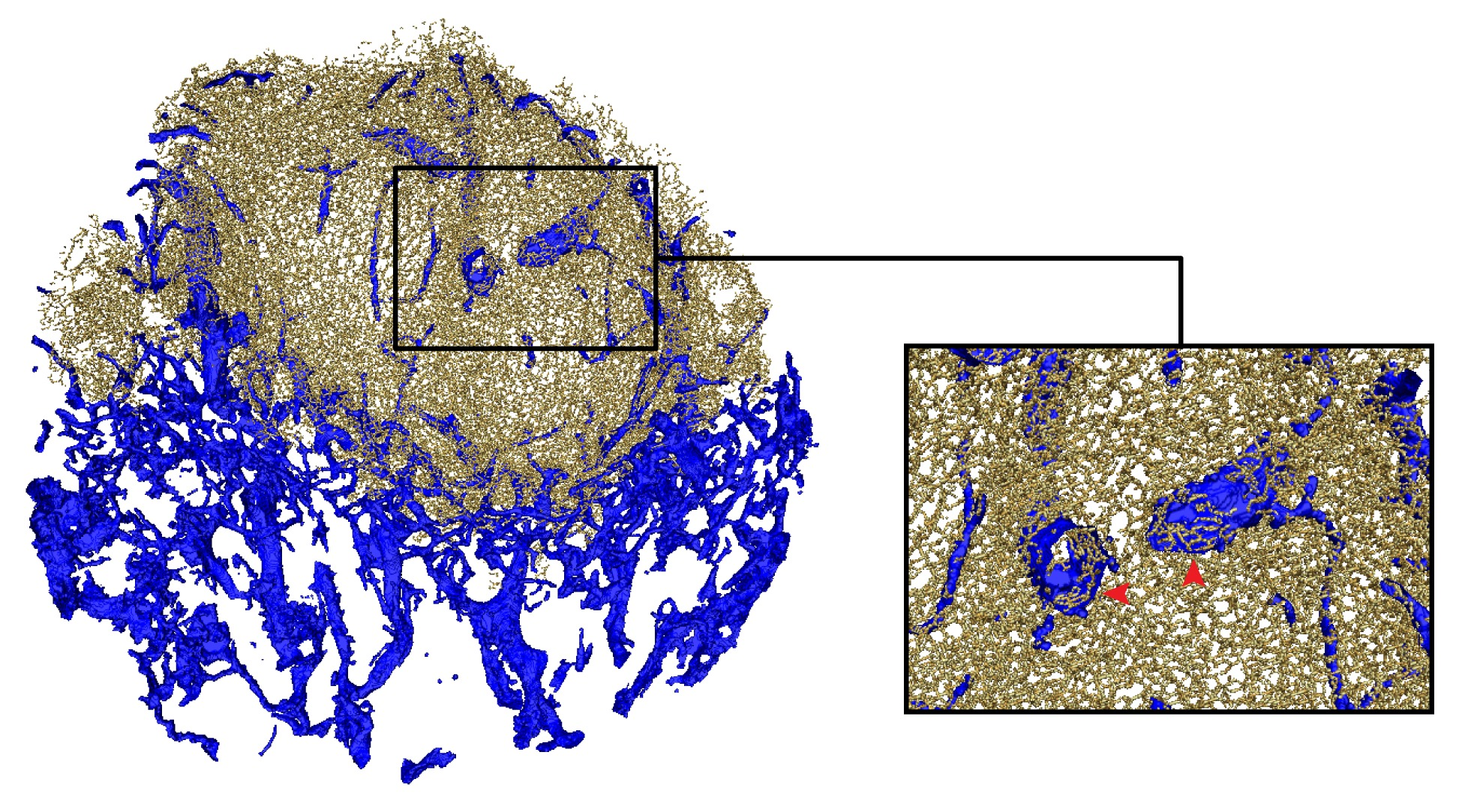

Supplement: S3 Fig — Related to Fig 2. A volume projection of the LN blood vasculature (blue) and conduit network (gold) demonstrates typical artifacts that occur during the segmentation of the fine conduit network around large blood vessels (close-up box). Here, the conduit network encloses the blood vasculature entirely and forms large tubes or sleeves that cannot be interpreted by the skeletonization algorithm, resulting in the creation of many short segments along the conduit sleeve (red arrowheads), hindering realistic analysis of the network at these locations. LN, lymph node. (TIF) [file pbio.3000486.s003.tif]

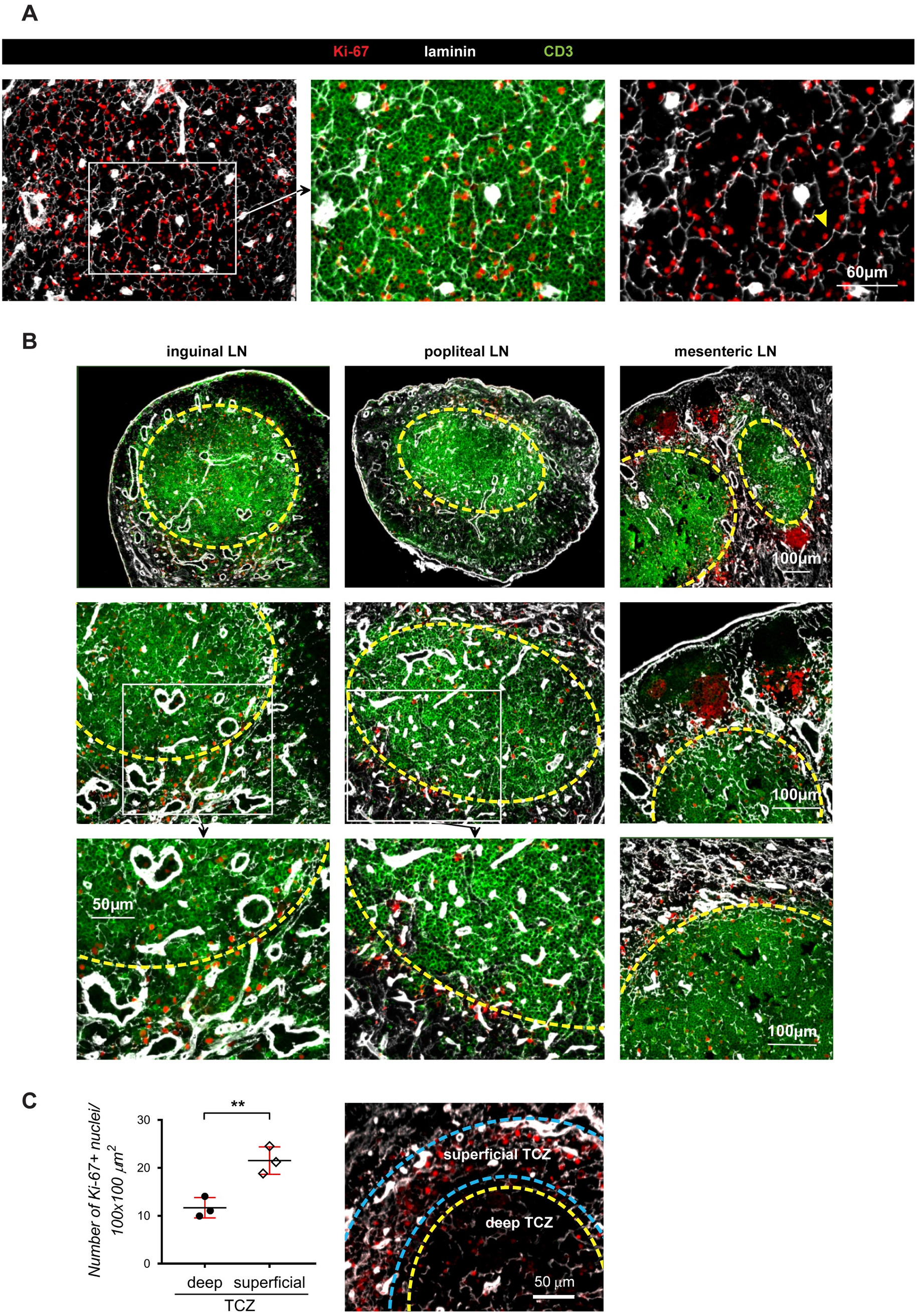

Supplement: S4 Fig — Related to Fig 3. Multicolor fluorescent images of immuno-labeled LN sections reveal a close association between Ki-67+ cells and laminin+ conduits in the CD3+ TCZ of an inguinal LN (yellow arrowhead, A). Overview of CD3+ TCZs (green) in inguinal, popliteal, and mesenteric LNs in which Ki-67+ cells can often be found close to the border of the TCZ (yellow dashed line, B). The number of Ki-67+ nuclei is significantly increased in the superficial TCZ compared with the deep TCZ (C). Ki-67 quantification was performed in ImageJ based on data from 3 independent experiments (each point represents either the deep or superficial zone from a mesenteric LN, N = 3). Plots show mean ± SD; **p < 0.01, Student t test. Values for each data point can be found in S1 Data. LN, lymph node; TCZ, T-cell zone. (TIF) [file pbio.3000486.s004.tif]
