# Supplementary material for: High-resolution 3D imaging and topological mapping of the lymph node conduit system
Source: PLoS Biol. 2019 Dec 19;17(12):e3000486. doi: 10.1371/journal.pbio.3000486 (PMC6922347; doi:10.1371/journal.pbio.3000486)
Supplement: Attachment — Submitted filename: Kelch et al PLOS Biol - response to reviewers 191021.docx [file pbio.3000486.s011.docx]

Kelch et al. 2019 Response to reviewers:

**Reviewer #1:** In the accompanying manuscript, Kelchen et al. present a high-resolution global overview on the conduit system in a murine lymph node, and such an overview has thus far been lacking in the field. The image quality and the videos are compelling. The authors provide useful quantification of their data sets, including a modelling of T cell migration patterns and the identification of conduits draining to defined areas within B cell follicles. Altogether, the data represent a valuable resource for modelling of immune responses and deepen our knowledge on the conduit system.

There are only minor comments from my side.

1.	Given the key role for EVIS in obtaining the images, the authors do not introduce this method in great detail, and one has to look in the Method section how the images were obtained.

Response to Reviewer #1, question 1:

We now added a short description of EVIS in the introduction:

“EVIS is a custom-build imaging platform that allows large tissue volumes (extending over several mm) to be imaged at sub-cellular resolution (down to 0.5 µm) by combining confocal microscopy with repeated rounds of precise sample trimming, and ultimately integrating overlapping images stacks into seamless 3D images. It is therefore particularly suited for imaging the fine conduit network across a tissue volume as big as a whole lymph node.”

Details about the voxel resolution of the image acquisition are provided in the results and are further comprehensively described in the methods section to satisfy readers with a particular interest in this imaging technique. We would also draw the reviewer’s attention to the previous publications regarding the EVIS method that are cited in the manuscript.

2.	Along this line, what is imaging throughput under the reported condition? For broader application of the technique, such information would be worth including in the manuscript.

Response to Reviewer #1, question 2:

Details regarding the imaging throughput are now provided in the methods section:

“Under these conditions, the imaging throughput using a single color channel was calculated to cover a volume of 1 mm^3^ at a voxel size of (1 um)^3^ within 1 day (8 hours). The dual-color image acquisition for the lymph node specimen presented here comprised 5 days in total. We are currently developing a new linear-scanning confocal imaging system, which acquires image data in parallel, with a speedup of around 100x.”

3.	How skewed is the distribution of Ki-67+ proliferating/recently proliferated cells (Figure S4)? This aspect has been overlooked in previous studies, so a quantification of the distribution would be of interest.

Response to Reviewer #1, question 3:

In response to the reviewer’s request, we quantified the Ki-67+ signal distribution in 2D immuno-labelled tissue sections using ImageJ and added the results to Figure S4. Compared to the deep T cell zone, the number of Ki-67+ nuclei per region can be nearly twice as high in the superficial T cell zone.

New paragraph under ‘Methods’:

“Ki-67 quantification

Analysis of the number of Ki-67+ nuclei per tissue region was performed in ImageJ. Individual images from immune-labeled LNs were scaled and thresholded before extracting the area and the number of particles from the Ki-67 channel within regions of interest, covering either the superficial or deep T cell zone based on the distribution of CD3e and laminin staining.”

Changes in the Figure legend S4 Fig:

“The number of Ki-67+ nuclei is significantly increased in the superficial TCZ compared to the deep TCZ (c). Ki-67 quantification was performed in ImageJ based on data from three independent experiments (each point represents either the deep or superficial zone from a mesenteric LN, N = 3). Plots show means ± SD, ** p < 0.01, Student’s t-test.”

4.	The accompanying videos would profit from a legend at the beginning such that one does not have to refer to the movie legend to assign fluorescent signals.

Response to Reviewer #1, question 4:

Legends have now been included in the video files.

5.	Figures 1h and i lack a scale bar.

Response to Reviewer #1, question 5:

The scale of the images in figure 1H and I are the same as Figure J but scale bars have been added to each image to improve clarity.

**Reviewer #2:** The study presents an efficient methodology for an organ-wide 3D imaging and quantitative analysis of the structural features of the entire LN conduit system. It can be regarded as a natural continuation/extension of the Authors’ previous published approach to systematically characterize the blood vessel system in a murine network. A unique confocal block-face imaging system (EVIS) is used to generate a comprehensive set of 3D images of LN regions supplied by conduits. The imaging-data-driven approach in conjunction with some original algorithms for the processing of the voxel-based 3D EVIS image of LN conduits enabled the construction of the complete 3D topological model of the conduit system. The study identified a number of fundamental properties of the conduit network. These include the structural parameters, i.e., the node degree-, edge length- and branching angle distributions, the distance to the nearest conduits, the heterogeneity in network density within the T cell zone, as well as the features relevant for the functional role of the conduit system in LN (the existence of follicular reservoirs, computer simulation-based estimates of cell motility characteristics).

The study opens new avenues for the development of spatially resolved computational models to examine quantitatively the role of the conduit system in LN fluid balance, transport of signaling molecules, and controlling the chemokine and cytokine fields affecting the lymphocyte motility and immune responses under various conditions. Overall, the results enable the quantification of the 3D conduit network parameters at exceptional resolution and precision. As such, they definitely will inspire further research in directions towards bioengineering of LNs, anatomically consistent mathematical modelling of the lymphatic system, and mechanistic understanding of the LN structure-function relationship.

The manuscript is clearly written and well-structured. The supplementary information is sufficient to grasp the power of the study.

I have no major concerns. Few minor comments:

1 Lines 274-276. There is no continuity in presenting the parts of Figure 4: the text jumps from a,b, to g,h.

Response to Reviewer #2, question 1:

Instead of jumping to G, H the text should reference Figure 4B-4D. This change has been applied.

2. Starting from Line 606 onwards: Model of T cell motility. How robust are the predictions with respect to the value of the coefficient of variation? Recent data indicate that the variability could be much larger, i.e. up 50 ~ 0.5 (for example, Sivapatham S, Ficht X, Barreto de Albuquerque J, Page N, Merkler D and Stein JV (2019) Initial Viral Inoculum Determines Kinapse-and Synapse-Like T Cell Motility in Reactive Lymph Nodes. Front. Immunol. 10:2086. doi: 10.3389/fimmu.2019.02086).

Response to Reviewer #2, question 2:

In response to the reviewer’s question, we tested a range of values for the coefficient of variation and for a typical case obtained a reduction of 5% in motility coefficient with a coefficient of variation of 0.5.

In order to keep the paper to a reasonable length we limited the amount of space given to results from the motility simulations. We are considering addressing remaining questions around motility in a separate paper devoted to the modeling to explore a number of issues.

3. Line 618: Could one justify the choice of the functional form (i.e. the forth power of the cosine of the turning angle) be justified by reference to data or previous modelling work?

Response to Reviewer #2, question 3:

The choice of the fourth power of the cosine was not based on data or previous models, it was simply a convenient functional form that conveys what we wanted – to encourage simulated cells to persist moving in the same direction, and to strongly discourage turns through more than 90 degrees.

**Reviewer #3**: Here, Kelch et al perform painstaking, high quality imaging of the lymph node (LN) conduits following labelling with injected fluourescent proteins. The imaging is excellent and although most of the data only confirm what has been previously published on the LN conduits, this study provides information about the conduits across an entire LN, as opposed to single slices. The data will benefit future mathematical modeling and simulation of the LN microenvironment.

The simulation of T cell migration present in this study adds an interesting element, although it is somewhat limited. The simulations are based on the premise that T cells remain in contact with the network of cells that form the conduit network. Most interesting is the difference in conduit density in the deep versus peripheral T cell zones. T cells migrating in the central T cell zone thus may not always contact the stromal/conduit network. The authors simulate T cell migration in both the deep and peripheral T cell zones, but the analysis assumes that the T cells contact the network to migrate. The findings from this simulation do not show any differences in migration of the T cells in either zone. However, it would be much better if the authors can simulate migration when the T cells do not need to migrate on the the network and compare the two zones. It seems to me that this may be more biologically relevant and the first time that this can be done because of the data generated in this study.

Response to Reviewer #3, question 1:

While we appreciate the intent of the reviewer’s suggestion, it is not clear to us how we would execute this suggestion with the tools available to us. The main intent of the motility simulations in the paper was to use the network geometry, in conjunction with the assumption of network-based motility, to see if the model could generate motility results consistent with what has been observed experimentally. This new network-informed model then allowed us to compare the simulated motility in two lymph node zones with different networks. Without using the network topology to constrain the motility, following the reviewer’s suggestion, we would have no differences between the two zones, negating any potential differences between modeled motility in these zones.

It is also worth noting that removing the network would require the development of a completely different model, which would of necessity be completely mathematical with no biological component underpinning it. While one can imagine such a model, implementing some version of a persistent random walk (as we have published in the past), this would not provide anything informative about the effects of different network topology on motility, since network topology had been stripped out from the model.

Finally, regarding reviewer #3’s statement that a network-independent model may be more biologically relevant, we would simply cite the wide acceptance that cells use the network to move around the LN (Bajenoff et al. (2006) Immunity 25, 989–1001, DOI 10.1016/j.immuni.2006.10.011; Hons et al. (2018) Nature Immunology 19, 606–616, https://doi.org/10.1038/s41590-018-0109-z ). We therefore submit that simulations of network-independent migration would be very unlikely to enhance the manuscript.

The authors also state a couple of times that the presence of extensive conduits in the B cell follicles was unexpected. Since others have published that conduits do serve the B cell follicles, and deliver soluble antigens to FDCs (ref 67 and others, as well as Nolte et al 10.1084/jem.20021801 in the spleen), these statements should be modified. The 3D imaging shown here does, however, improve our understanding of what these structures look like, in particular the FDC structures.

Response to Reviewer #3, question 2:

We have modified the respective passages (line 267 and 271) to satisfy the reviewer’s comment:

“EVIS imaging of WGA-perfused LNs led to the intriguing observation of distinctive tracer accumulations inside B cell follicles. (…)

However, we also observed distinctive WGA tracer accumulations within the B cell regions, appearing as discrete multilobular spaces reminiscent of ‘honeycombs’ that are connected to the SCS and each other via follicular conduits (Fig 4 a, b, S4 Video), occasionally aggregating into larger contiguous cavities spanning the entire B cell follicle.”

In principle, several other reports do indicate tracer distributions in the B cell follicle, and in particular the transition from conduits to FDCs near the SCS as the reviewer notes. However, we hope our imagery will provide some clarity on the true extend of tracer distribution within the entire B cell region and make it easier to appreciate the differences between collagen-supported conduits and FDC reservoirs.

Minor comment: on line 196, page 12: simulated and not stimulated?

Response to Reviewer #3, question 3:

Thank you for pointing out this error, it should indeed say ‘simulated’. The passage has been modified accordingly.
